# Attitudes and behaviour towards psychotropic drug prescribing in Swedish primary care: a questionnaire study

**DOI:** 10.1186/s12875-018-0885-4

**Published:** 2019-01-05

**Authors:** Staffan A. Svensson, Tove M. Hedenrud, Susanna M. Wallerstedt

**Affiliations:** 1 Närhälsan Hjällbo GP Practice, Bergsgårdsgärdet 89B, SE-424 32, Angered, Sweden; 2 Department of Public Health and Community Medicine, Sahlgrenska Academy, University of Gothenburg, Gothenburg, Sweden; 3 Department of Pharmacology, Sahlgrenska Academy, University of Gothenburg, Gothenburg, Sweden; 4 Department of Clinical Pharmacology, Sahlgrenska University Hospital, Gothenburg, Sweden

**Keywords:** Prescribing, Psychotropic drugs, Primary health care, Questionnaire

## Abstract

**Background:**

The prescribing of psychotropic drugs, i.e. antidepressants, sedatives (anxiolytics, hypnotics), and antipsychotics is considerable and a large proportion is prescribed by general practitioners (GPs). There are concerns about dependency and medicalisation, and treatment decisions in psychiatry may appear arbitrary. Increased knowledge of GPs’ opinions on the prescribing of psychotropics may lead to more rational use of these drugs. We aimed to quantify GPs’ attitudes, beliefs and behaviour towards various aspects of psychotropic drug prescribing.

**Methods:**

A questionnaire was distributed to physicians in all 199 GP practices in Region Västra Götaland, Sweden. The questions concerned determinants of psychotropic drug prescribing that had been identified in a previous, qualitative study.

**Results:**

Questionnaires from 516 physicians (64% of whom were specialists in family medicine, 21% interns in family medicine, 15% others) at 152 GP practices (59% of which were state owned, 72% in an urban area, with a median of 7808 registered patients) were returned (estimated response rate: 48%). A majority – 62% – of GPs found it easier to start prescribing psychotropic drugs than to stop (95% confidence interval, 57%, 66%) vs. 8% (6%, 10%). Most GPs considered psychotherapy more suitable than psychotropic drugs in cases of mild psychiatric disease: 81% (77%, 84%) vs. 4% (3%, 6%). The problems treated with psychotropic drugs were considered to be mostly socioeconomic, or mostly medical, by similar proportions of physicians: 38% (34%, 42%) vs. 40% (36%, 45%). GPs were on average satisfied with their levels of antidepressant and sedative prescribing in relation to medical needs. More GPs regarded their prescribing of antipsychotics as being too low rather than too high: 33% (28%, 39%) vs. 7% (4%, 10%).

**Conclusions:**

This study illustrates the complexities of psychiatric drug treatment in primary care and identifies potential drivers of increased prescribing of psychotropics. The manifold factors, medical and non-medical, that affect prescribing decisions may explain a sense of arbitrariness surrounding psychotropic drug treatment. This notwithstanding, GPs seem mostly content with their prescribing.

**Electronic supplementary material:**

The online version of this article (10.1186/s12875-018-0885-4) contains supplementary material, which is available to authorized users.

## Background

The global burden of mental illness is considerable and has remained stable over recent decades [1]. In Sweden, psychiatric disease is the most common reason for sick leave among women, and the second most common among men [2]. Many patients with psychiatric problems are treated in primary health care, and psychotropic drugs are an important treatment modality for psychiatric disorders. The use of psychotropic drugs is prevalent internationally [3–5] as well as in the Swedish context. Data from the Swedish Prescribed Drug Register [6] shows that in 2016, 96 out of 1000 inhabitants collected at least one prescription of antidepressant drugs, representing an increase of 21% since the year 2006 [7]. Corresponding figures for anxiolytics were 57 prescriptions (increase of 9%), for hypnotics 77 (decrease of 3%), and for antipsychotics 15 (decrease of 8%) [7].

In many countries, general practitioners (GPs) are responsible for the bulk of psychotropic prescribing. In Sweden, some 70–80% of all psychotropic drugs for the elderly are prescribed through primary care [8]. In neighbouring Norway, GPs initiate 73% of antidepressants and 58% of antipsychotics in the general population [9].

The high prevalence of psychotropic drug use evokes mixed feelings among GPs. In the literature, decisions concerning the prescribing of psychotropics in primary care are often described as sensitive and problematic [10, 11]. Although these drugs are considered important therapeutic instruments, there are concerns about immediate and delayed side-effects, dependency, abuse, withdrawal symptoms, and a general sense of unease about the medicalisation of common life situations [12, 13]. While there is seldom any hesitation about treating clear-cut cases, e.g. of major depression, the clinical presentation in primary care is more commonly one of ambiguous psychiatric symptoms, frequently in combination with unexplained somatic symptoms. Moreover, there is much intertwining of medical and socioeconomic problems [11, 14, 15].

Given this complex and challenging setting, it is perhaps not surprising that GPs sometimes report being reluctant to prescribe a psychotropic drug but nevertheless find themselves doing so in the absence of better options [13, 16, 17]. Using qualitative methods, we have previously explored factors behind psychotropic drug prescribing in primary care [10]. Among the themes that emerged were: the social determinants of psychiatric problems, views on the connection between the price and the effect of drugs, prescriptions initiated by (or “inherited” from) other physicians, the influence of prescribing technology, and the choice between psychotherapy and drug treatment [10]. The aim of the present study was to quantify GPs’ attitudes, beliefs and behaviour towards some of these factors.

## Methods

A descriptive questionnaire study was performed among physicians in GP practices in Region Västra Götaland. The study protocol was approved by the Regional Ethical Review Board in Gothenburg, Sweden (reference number: 777–10). The target population was all physicians working, as regulars or locums, at a GP practice in the region. The term “GP” will be used here in the wider sense of “a physician working in primary care”, i.e. not necessarily a specialist in family medicine (unless otherwise indicated). Region Västra Götaland is located in the south west of Sweden and has 1.6 million inhabitants (17% of the Swedish population), across a mixed rural and urban area. At the time of data collection, there were 199 GP practices in the region.

In September 2012 we sent envelopes containing cover letters, paper questionnaires for completion by physicians, and pre-paid return envelopes to the heads of all GP practices in the region, with a letter asking the head to distribute these to all physicians currently working in the practice. The number of questionnaires in each envelope was based on an estimate of the number of physicians at each practice, plus some extra copies. A reminder was sent to the practice heads three weeks later.

The cover letter to the physicians explained the purpose of the study and pointed out that the questionnaires had corner marks identifying the GP practice, which could be removed to make them unidentifiable. Ample time was allowed for replies, as interns in family medicine are sometimes absent on long clinical rotations. Most replies arrived within 1 month, the last one arriving in March 2013.

The questionnaire was based on results from a focus group study exploring factors that affect the prescribing of psychotropic drugs in primary care [10]. A draft was tested for face and content validity on physicians not in the target population. The final questionnaire had 20 single-response questions and fitted on two sides of a single A4 sheet – see the Swedish original in Additional file 1 and an English translation in Additional file 2. The questionnaire began by defining psychotropic drugs as antidepressants, anxiolytics/hypnotics and antipsychotics. In what follows, we use the term *sedatives* to denote the largely overlapping groups of anxiolytics and hypnotics. The first 10 questions assessed the physician’s attitudes, beliefs and behaviours towards psychotropic drugs, with ordinal scales graded 1–5 (questions 1–9) or 1–3 (question 10). The following three questions referred to the physician’s rating of his/her prescribing of antidepressants, sedatives and antipsychotics, with ordinal scales graded 1–5 and a “not applicable” option for respondents who had not prescribed from that category. Questions 14–20 concerned the physician’s education, demographics and workplace characteristics.

Data from the returned questionnaires were entered into a custom database by a secretary. Problematic entries were checked by the authors and inconsistencies were treated conservatively (for example, markings between boxes were extrapolated to the one nearest the middle).

The GP practices were categorised according to disease burden, socioeconomic context, ownership, and type of district in which they were located. The Johns Hopkins Adjusted Clinical Groups (ACG) System [1] was used as a measure of the disease burden of the practice population, and the Care Need Index (CNI) [2] as a measure of the population’s socioeconomic determinants of health care need. Both are used for remuneration purposes and were obtained from the Primary Healthcare Office, as were data on ownership (private/state) and the number of patients registered in each practice. Eurostat’s three levels of urbanisation [20] were used to classify practices as either urban (combining the levels *densely* and *intermediately* populated), or rural (the level *thinly* populated).

### Statistics

Data were analysed using the statistical package R-3.2.2 [21]. The analyses were primarily descriptive. ACG and CNI figures were divided into quartiles 1, 2–3 and 4, with the upper quartile (1) indicating the highest disease burden (ACG) and the highest expected health care need (CNI). Confidence intervals (CI) for proportions were calculated using the Wilson method. A binomial logistic regression was performed to explore differences between GP practices with 0 vs. ≥1 replies, with urban/rural location, private/state ownership and the number of registered patients as covariates. Spearman correlations between the ordinal scale questions were calculated to determine whether a multivariate analysis was worthwhile.

## Results

**Fig. 1:**
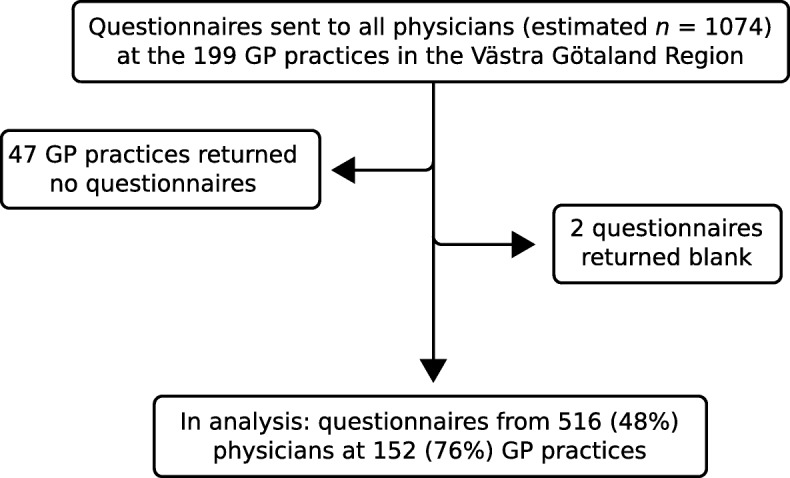
Participant recruitment

Questionnaires from 516 physicians, working in 152 GP practices, were included in the analysis (Fig. 1). We could not calculate an exact response rate, as there was no accurate list of the number of physicians working in primary care. An estimate from the Primary Healthcare Office, however, put the number of physicians at 1074, of which approximately 65% were specialists and 30% interns in family medicine. Using this estimate, the response rate was 48%.

The responding physician’s work place was unknown in three cases, due to the identification mark having been torn off the questionnaire. Comparison of GP practices with ≥1 vs. 0 returned questionnaires showed that responding practices had more registered patients and were more often state-owned and located in urban areas (Table 1). In the logistic regression, however, only the number of registered patients remained significantly associated with response status (data not shown).

**Table 1:** Characteristics of GP practices with ≥1 (*n* = 152) and 0 (*n* = 47) returned questionnaires

		≥1 returned questionnaires	0 returned questionnaires
Registered patients*		7808 (5452–10,708)	5884 (4306–8971)
ACG^a^	Upper quartile	38 (25)	12 (26)
	Quartiles 2–3	75 (49)	24 (51)
	Lower quartile	39 (26)	11 (23)
CNI	Upper quartile	37 (24)	13 (28)
	Quartiles 2–3	76 (50)	23 (49)
	Lower quartile	39 (26)	11 (23)
Ownership	State	90 (59)	23 (49)
	Private	62 (41)	24 (51)
Area	Urban	110 (72)	29 (62)
	Rural	42 (28)	18 (38)

^*^Median number of patients registered at practice (interquartile range). ^a^Number (%) of GP practices. ACG = Adjusted Clinical Groups [18], CNI = Care Need Index [19]

Among the responding physicians, 250 (49%) were female and 462 (91%) were at their regular workplace. A total of 325 (64%) were specialists in family medicine, 108 (21%) were interns in family medicine, 37 (7%) worked under a provisional medical registration, and 39 (8%) did not fit into any of these categories. The mean number of years since medical registration was 17.5 (range 0–44). In all, 495 (98%) could refer patients for psychotherapy at their practice, and 42 (8%) had on-site access to a psychiatrist. Among the respondents, 263 (52%) had attended a pharmaceutical industry information session over the last three months.

Table 2 presents the GPs’ responses concerning attitudes, beliefs, and behaviour towards psychotropic drugs. A majority of GPs found it somewhat easier, or much easier, to start prescribing psychotropic drugs than to stop prescribing them: 62% (95% CI: 57%, 66%) vs. 8% (6%, 10%). More GPs found it somewhat or very difficult, rather than easy, to change a colleague’s prescription of a psychotropic drug: 38% (34%, 43%) vs. 25% (21%, 29%).

**Table 2:** Questions about physicians’ attitudes, beliefs and behaviour towards psychotropic drug prescribing. All questions except the last had five possible replies: the endpoints are shown. Figures are n (% of non-missing) for replies, and n (% of all respondents, *n* = 516) for missing

Question	Endpoints			Replies			Missing
		1	2	3	4	5	
1 Which do you find easier: starting or stopping prescribing psychotropics?	Starting much easier (1) Stopping much easier (5)	81 (16)	237 (46)	158 (31)	31 (6)	9 (2)	0 (0)
2 In your experience, are the problems you treat with psychotropics more social/economic, rather than medical?	Yes, very often (1) No, very rarely (5)	23 (4)	173 (34)	110 (21)	154 (30)	54 (11)	2 (0)
3 How do you feel about changing a colleague’s psychotropic prescription?	Very difficult (1) Very easy (5)	25 (5)	173 (34)	189 (37)	108 (21)	20 (4)	1 (0)
4 Do you believe your patients perceive expensive psychotropics as being more or less effective than cheap ones?	Much more effective (1) Much less effective (5)	25 (5)	152 (30)	319 (63)	9 (2)	3 (1)	8 (2)
5 In your experience, how do health care staff behave towards patients who use psychotropics, compared to their behaviour towards other patients?	Much better (1) Much worse (5)	0 (0)	14 (3)	362 (71)	132 (26)	5 (1)	3 (1)
6 When working in primary care, do you prescribe psychotropics that have recently appeared on the market?	Yes, very often (1) No, very rarely (5)	2 (0)	16 (3)	39 (8)	202 (39)	257 (50)	0 (0)
7 In your opinion, are new psychotropics more effective than older ones?	Much more effective (1) Much less effective (5)	6 (1)	167 (33)	308 (62)	17 (3)	1 (0)	17 (3)
8 If your patient has dose-dispensed drugs, do you ever repeat several prescriptions at the same time, without assessing each individual prescription?	Yes, very often (1) No, very rarely (5)	14 (3)	114 (22)	66 (13)	180 (35)	138 (27)	4 (1)
9 In mild psychiatric disease, what kind of treatment do you consider most suitable: psychotherapy (PT) or psychotropic drugs (PD)?	PT much more suitable (1) PD much more suitable (5)	192 (38)	213 (42)	78 (16)	16 (3)	4 (1)	13 (3)
		1	2	3			
10 Keeping symptoms constant, how much do you suppose psychotropic prescribing varies between different GP practices?	It varies considerably (1) It varies negligibly (3)^a^	224 (44)	251 (50)	29 (6)			12 (2)

^a^The last question had 3 possible replies

There was a biphasic distribution in GPs’ reported behaviour when repeating multi-dose dispensed medications: 62% (58%, 66%) renewed “in bulk” quite rarely or very rarely, whereas 25% (21%, 29%) reported doing so quite often or very often. Regarding the variation between GP practices in prescribing psychotropic drugs, given equal symptoms, more GPs believed it varied considerably rather than negligibly: 44% (40%, 49%) vs. 6% (4%, 8%).

A large majority of GPs considered psychotherapy somewhat more, or much more, suitable than psychotropic drugs in mild psychiatric disease: 81% (77%, 84%) vs. 4% (3%, 6%). Opinions were divided about the nature of problems treated with psychotropic drugs: 38% (34%, 42%) considered them mostly socioeconomic, while 40% (36%, 45%) considered them mostly medical. A majority of GPs believed health care staff behaved in a similar way towards patients who use psychotropic drugs, in comparison to their behaviour towards non-users. Among those GPs who did not believe this, most considered staff behaviour somewhat worse or much worse, rather than better: 27% (23%, 31%) vs. 3% (2%, 5%).

Three questions concerned new and expensive drugs in relation to old and cheap ones. Most GPs considered new and old psychotropic drugs equally effective, but an appreciable proportion saw new drugs as somewhat, or much more, effective than old ones: 35% (31%, 39%) vs. 4% (2%, 6%). Similarly, most believed that *patients* felt there was no connection between drug price and effectiveness. Among those with a differing view, however, a majority believed that patients considered expensive psychotropics to be somewhat, or much more, effective than cheap ones: 35% (31%, 39%) vs. 2% (1%, 4%). As for their actual behaviour, most GPs reported that they quite rarely, or very rarely, prescribed psychotropics that have recently emerged on the market, rather than quite often or very often: 89% (86%, 91%) vs. 3% (2%, 5%).

Most GPs viewed their prescribing of psychotropic drugs as appropriate, in relation to their patients’ medical needs (Table 3). The estimates of antidepressant and sedative prescribing were evenly distributed around the center option representing an adequate amount. There was more spread to the side options of “somewhat high” and “somewhat low” for sedatives than for antidepressants. Almost all GPs reported having prescribed antidepressants and sedatives in the last three months, whereas nearly half (45%) reported not having prescribed antipsychotics. Among those who had, a larger number rated their level of prescribing as somewhat low or very low, rather than high: 33% (28%, 39%) vs. 7% (4%, 10%).

Correlations between the ordinal scale items (i.e. the variables in Tables 2 and 3) were low (absolute mean 0.08, maximum 0.31), and we therefore refrained from multivariate analysis of these variables.

**Table 3:** Questions about prescribing. Replies to the question “Over the last three months, how would you describe the level of your prescribing of [type of psychotropic], in relation to the medical needs of your patients?”. Figures are n (% of prescribers) for replies Very high through Very low; n (% of non-missing) for non-prescribers, and n (% of all respondents, *n* = 516) for missing

			Replies				Missing
	Very high	Somewhat high	Neither one nor the other	Somewhat low	Very low	Have not prescribed	
Type of psychotropic							
Antidepressants (*n* = 498^*^)	13 (3)	61 (12)	369 (74)	51 (10)	4 (1)	10 (2)	8 (2)
Anxiolytics/hypnotics (*n* = 502^*^)	12 (2)	100 (20)	292 (58)	86 (17)	12 (2)	6 (1)	8 (2)
Antipsychotics (*n* = 279^*^)	7 (3)	12 (4)	168 (60)	50 (18)	42 (15)	227 (45)	10 (2)

^*^Number of prescribers

## Discussion

### Summary of main findings

This study shows that Swedish GPs find it easier to start than to stop prescribing psychotropic drugs and that there is some reluctance to alter other physicians’ prescriptions. A considerable share of GPs believe between-practice variation in psychotropic drug prescribing is high, given equal symptoms. Similar proportions of GPs believe patients take psychotropic drugs for reasons that are mostly medical, or mostly socioeconomic, respectively. Although few GPs report actually prescribing new drugs, there is a tendency to believe, or to believe that patients think, that new and expensive drugs are more effective than older and cheaper ones. GPs are on average satisfied with their level of prescribing of antidepressants and sedatives. The prescribing of antipsychotics, on the other hand, is more often rated as too low than too high, and only about half the GPs report prescribing them.

### Attitudes, beliefs and behaviours

Among the GPs in this study, six out of ten found it easier to start than to stop prescribing psychotropic drugs, whereas the opposite was true for only one in ten. The ease of starting and stopping a drug may be linked to the tendency of that drug to cause dependency, making sedatives of the benzodiazepine type particularly difficult to stop [12, 17]. One Dutch-Swedish study found, for example, that two thirds of patients who started taking benzodiazepines were still taking them one year after initial prescription, and one third were still taking them after eight years [22].

Of course, many factors other than the drug’s pharmacological properties influence prescribing decisions. Commonly, GPs are faced with the request to repeat a prescription originally issued by another physician, a situation that poses a particular set of problems. In the present study, GPs more frequently found it difficult rather than easy to change a colleague’s prescription. The assessment of another physician’s reason for prescribing may be difficult because of limited information in the medical records [23]. Moreover, when a prescription is “inherited” from another physician, the receiving GP is likely to consider some of the responsibility remains with the initiator [11]. This tendency may be more pronounced for problematic drugs such as benzodiazepines and opioids, where assuming full responsibility may imply that the GP should take on the demanding and sometimes thankless task of trying to reduce dosages [11].

Psychiatric drug therapy is often seen as being more arbitrary than its somatic counterpart, a finding we highlighted in a paper entitled “Psychiatry is not a science like others” [10]. In the present study, about half of the respondents believed there was considerable variation in psychotropic prescribing (for equal symptoms) between GP practices; the other half was undecided and a few considered variation to be negligible. These findings are in agreement with variations in psychotropic prescribing practices nationally, with higher use of antidepressant and sedative drugs in western Sweden and higher use of antipsychotic drugs in the north [7]. Internationally, there are also significant differences in the choice and volume of psychotropic drugs prescribed between regions and nations, with more frequent use among women, the elderly, and the socially deprived [3–5]. Even accounting for these factors, however, much variation remains unexplained and is sometimes put down to the “diverse prescription habits of physicians” [4].

In the present study, the GPs were overwhelmingly in favour of using psychotherapy rather than psychotropic drugs for mild psychiatric disease, a finding that may indicate that Swedish GPs define “mild” disease in terms of “not needing psychotropic drugs”. The high availability of psychotherapy in Swedish primary care, illustrated by our finding that virtually all respondents had access to psychotherapy at their practice, makes referral feasible. Internationally, on the other hand, a scarcity of psychotherapists is often cited as a factor that contributes to high levels of prescribing of psychotropic drugs in primary care [15–17].

The question concerning the nature of problems treated with psychotropic drugs divided the GPs into two equally sized camps. Eight out of ten respondents saw these problems as either predominantly socioeconomic or predominantly medical, and only two in ten chose the in-between option. The framing of a patient’s condition is, indeed, often contentious in general practice: does the patient actually suffer from a psychiatric disease, or are the symptoms more properly viewed as manifestations of his/her difficult life situation [13, 24]? Making a distinction between medical and socioeconomic factors as the cause of ill health may be difficult in any health care setting. It is, however, arguably more difficult in primary care, as GPs treat patients with milder symptoms and may also be more aware of patients’ circumstances through long-established contact and knowledge of other family members [15, 25].

Most respondents, seven out of ten, believed health care staff behaved similarly towards users and non-users of psychotropic drugs. Nevertheless, almost all of the remainder believed that behaviour towards users of these drugs was worse than that towards other patients. In terms of stigmatisation of the mentally ill, the secular trend is probably one of decreasing stigma [10, 13].

Six out of ten GPs were neutral about the association between a psychotropic drug’s newness and its perceived efficacy. Three out of ten believed new drugs were more effective than old ones. These proportions were very similar to the GPs’ estimates of *patients’* opinions about efficacy in relation to expensiveness. Whereas some GPs do believe new and expensive drugs are more effective [10], most research indicates that GPs place little emphasis on cost in treatment decisions [26]. Internationally, physicians have been found to make largely inaccurate estimates of medicine prices [27, 28]. Very few GPs in our study claimed to actually prescribe new psychotropic drugs, a finding in line with previous research indicating that Swedish GPs tend to follow the therapy recommendations issued by Drugs and Therapeutic Committees [29].

Decisions about drug therapy may be influenced by prescribing technology. Multi-dose dispensing, where a patient’s drugs are automatically dispensed in plastic bags corresponding to each instance of administration, is common among the elderly in Sweden [30]. At the time of the study, the electronic interface for prescribing multi-dose dispensed drugs had a function for repeating all the patient’s drugs by a single click of the mouse [10]. In our study, the question about dose-dispensed drugs and *en masse* renewal, i.e. without individual consideration of each medication, yielded mainly negative answers. However, a quarter of the GPs claimed to do this more or less often. Consistent with this finding, other studies have shown that the drug regimens of patients with multi-dose dispensing are reassessed less frequently [31], and that patients with normal prescriptions have more appropriate drug regimens than those with multi-dose dispensing do [23, 32]. Concerns about the safety of the repeat-all function finally led to its removal.

The GPs in the present study were, overall, content with their prescribing levels in relation to medical needs: seven out of ten were satisfied with their prescribing of antidepressants, and six out of ten with their prescribing of sedatives and antipsychotics. The latter category stood out, however, in two respects. First, whereas almost all GPs had prescribed from the first two drug classes, only about half claimed to have prescribed antipsychotics recently. Second, whereas the distribution of dissatisfied prescribers was symmetrical for antidepressants and sedatives, it was highly skewed towards the “too low” side for antipsychotics. It thus appears that GPs view antipsychotics as a drug group that is rarely prescribed and somewhat underused. Prescription rates of antipsychotics are, as noted in the introduction, indeed much lower than those of the other main drug categories. A potential explanation for the unexpected finding of perceived underuse may be that antipsychotics may be interpreted semantically as “drugs for psychosis”, thereby evoking a context wherein patients are often reluctant to comply with therapy and prone to use too little medication.

### Drivers of increased prescribing

As seen in the introduction, the prescribing of psychotropic drugs is rising in Sweden. The increase has, however, largely been confined to antidepressants, mirroring the expanding use of drugs from this category in several European countries [3]. Meanwhile, the international trend for the prescribing of sedatives is mixed [4], whereas that of antipsychotics seems to be rising [5]. Keeping this in mind, what are the implications of our findings for the wider aim of the project – understanding the determinants of psychotropic drug prescribing in primary care?

First, the finding that GPs are satisfied with their level of prescribing in spite of high (and rising) levels of antidepressant use, may indicate that this drug category is seen as safe and useful. Granting that many GPs feel uneasy about increasing levels of antidepressant prescribing [13], the dominant attitude may still be that antidepressants are comparatively innocuous [10, 15]. It may be hypothesised that high levels of antidepressant prescribing are tolerated, as long as the level of sedatives prescribed is under control.

Second, we show that GPs find it easier to start than to stop prescribing psychotropic drugs. Importantly, we did not ask if this led to GPs actually starting prescribing drugs more often. Nevertheless, if acted upon, this inclination would tend to inflate prescribing rates over time. Moreover, the GPs indicated some reluctance to change a colleague’s prescription, which would similarly favour increased prescribing, if acted upon. Requests for repeating other physicians’ prescriptions may be problematic for reasons already highlighted; additionally, it is often expected that a drug regimen initiated by a psychiatrist will be continued after referral back to primary care [10]. More generally, changing or refusing to repeat another physician’s prescription may be construed as a sign of disrespect towards that colleague, and may therefore be avoided.

Third, touching on the previous point, there are a number of “not strictly medical” considerations that are clearly of importance in understanding the mechanisms of psychotropic prescribing in primary care. Patients’ expectations are a major factor, and GPs often report that patients seek simple solutions to complex psychosocial problems [10, 13]. In line with this, we found that many GPs regard socioeconomic factors as relevant for psychotropic prescribing. Whatever the GP holds to be true about a symptom being “medical” or not may, however, be of less immediate significance than the practical need of getting through a day’s work. Thus, a high work load, lack of alternative therapies and a general sense of being overwhelmed by patients’ predicaments may lead to the prescription of psychotropics as a coping mechanism [16]. Awareness of this from personal experience may be one reason why GPs consider psychiatric drug treatment to be somewhat arbitrary.

### Strengths and limitations

The high number of respondents is a strength of the present study. Nevertheless, the response rate is a limitation; we do not know if non-responders’ replies would have differed from those obtained in the study. However, the response rate is similar to that in many other studies based on questionnaires sent to physicians (e.g. [12, 17]), and the distribution of specialists and interns among the responders was similar to that in the overall population. Characteristics of responding and non-responding GP practices were also similar in terms of location and ownership, after controlling for the number of registered patients. Practices with more registered patients were more likely to respond; an expected finding as larger practices usually have more physicians. Further, the returned questionnaires were largely complete, with little missing data.

A limitation of the study is that a 20-item questionnaire is unable to explain more than a small part of a complex phenomenon such as psychotropic drug prescribing. A further limitation of the study is that the questionnaire used was not extensively validated. In addition, we mainly used the umbrella term “psychotropic drugs” in the questionnaire, after presenting the three main psychotropic drug categories. This choice reflects our previous use of this term [10] and is based on the view that psychotropic medications have many common features. In addition, the labels attached to psychiatric drug categories are poorly matched to the drugs’ actual effect and clinical use. Antidepressants, for example, are arguably more useful in anxiety disorders than in depression [33]. Further, in contrast to much other research, we did not restrict the questions to patients in a certain age range or with a specific psychiatric disorder. This approach generated knowledge with a broad scope, which we believe is relevant to primary care.

Finally, we consider it a strength that the input of all physicians working in primary care was sought, including temporary staff, and that the questionnaire we used was based on our previous research using qualitative methodology [10]. Future research could focus on integrating qualitative and quantitative components, using a mixed methods design, with the aim of further elucidating physicians’ rationales for decision making about psychotropic drugs.

## Conclusions

In conclusion, our results highlight some of the complexities surrounding the prescribing of psychotropic drugs in general practice. The findings may be useful for physicians, policy-makers and researchers who endeavour to understand patterns of prescribing. Although some sense of arbitrariness may be inevitable when it comes to psychotropic drug therapy, the issue of responsibility for psychotropic prescribing, particularly long-term, merits more attention. Further, the fact that most GPs were satisfied with their levels of prescribing may require clarification, as this may indicate both self-delusion and/or a supreme insight into the patients’ circumstances.

## Additional files

Additional file 1: Questionnaire. In Swedish, as sent to participants. (PDF 881 kb)

Additional file 2: Questionnaire translation. With names of variables, original text and English translation. (PDF 52 kb)

## Abbreviations

ACG: Adjusted Clinical Groups
CNI: Care Need Index
GP: General practitioner
