# Supplementary material for: Attitudes and behaviour towards psychotropic drug prescribing in Swedish primary care: a questionnaire study
Source: BMC Fam Pract. 2019 Jan 5;20:4. doi: 10.1186/s12875-018-0885-4 (PMC6321667; doi:10.1186/s12875-018-0885-4)
Supplement: Supplementary file 1 — Questionnaire. In Swedish, as sent to participants. (PDF 881 kb) [file 12875_2018_885_MOESM1_ESM.pdf]

Vad tycker du?

I intervjuer med vårdcentralsläkare om psykofarmaka (antidepressiva läkemedel, lugnande/sömnmedel och antipsykotika) fick vi en rad förslag på faktorer som kan påverka förskrivningen. Vi skulle vilja veta din åsikt om dessa, och hur du ser på din egen förskrivning!

---

1 Vad tycker du är lättast: att sätta **in** eller sätta **ut** psykofarmaka?

- Sätta in mycket lättare ..... ☐
- Sätta in något lättare .... ☐
- Ingen skillnad .... ☐
- Sätta ut något lättare .... ☐
- Sätta ut mycket lättare ..... ☐

2 Upplever du att de problem du behandlar med psykofarmaka är sociala/ekonomiska, snarare än medicinska?

- Ja, mycket ofta .... ☐
- Ja, ganska ofta .... ☐
- Varken/eller .... ☐
- Nej, ganska sällan .... ☐
- Nej, mycket sällan .... ☐

3 Hur tycker du det är att ändra en kollegas ordination av psykofarmaka?

- Mycket svårt .... ☐
- Ganska svårt .... ☐
- Varken/eller .... ☐
- Ganska lätt .... ☐
- Mycket lätt .... ☐

4 Tror du att dina patienter uppfattar dyra psykofarmaka som mer eller mindre effektiva än billiga?

- Mycket mer effektiva .... ☐
- Något mer effektiva .... ☐
- Ingen skillnad .... ☐
- Något mindre effektiva .... ☐
- Mycket mindre effektiva .. ☐

5 Hur upplever du att sjukvårdspersonal bemöter patienter som står på psykofarmaka, jämfört med andra patienter?

- Mycket bättre .... ☐
- Något bättre .... ☐
- Ingen skillnad .... ☐
- Något sämre .... ☐
- Mycket sämre .... ☐

6 När du arbetar i primärvården, skriver du då ut psykofarmaka som är nya på marknaden?

- Ja, mycket ofta .... ☐
- Ja, ganska ofta .... ☐
- Varken/eller .... ☐
- Nej, ganska sällan .... ☐
- Nej, mycket sällan .... ☐

7 Anser du att nya psykofarmaka överlag är mer eller mindre effektiva än gamla?

- Mycket mer effektiva .... ☐
- Något mer effektiva .... ☐
- Ingen skillnad .... ☐
- Något mindre effektiva .... ☐
- Mycket mindre effektiva .. ☐

8 Om din patient har Apodos, händer det då att du förnyar flera recept samtidigt utan att värdera varje enskild ordination?

- Ja, mycket ofta .... ☐
- Ja, ganska ofta .... ☐
- Varken/eller .... ☐
- Nej, ganska sällan .... ☐
- Nej, mycket sällan .... ☐

9 Vid lindrig psykiatrisk sjukdom, vilken behandlingsform anser du mest lämplig: samtalsterapi (S) eller psykofarmaka (P)?

- S mycket lämpligare .... ☐
- S något lämpligare .... ☐
- Ingen skillnad .... ☐
- P något lämpligare .... ☐
- P mycket lämpligare .... ☐

Vad tycker du? (forts)

10 Vid samma symtombild, hur mycket tror du förskrivningen av psykofarmaka varierar mellan olika vårdcentraler?

- Den varierar mycket .... ☐
- Den varierar något .... ☐
- Den varierar försumbart .. ☐

11 Under de tre senaste månaderna, hur anser du att din förskrivning av **antidepressiva läkemedel** varit i förhållande till dina patienters medicinska behov?

- Mycket hög .... ☐
- Ganska hög .... ☐
- Varken/eller .... ☐
- Ganska låg .... ☐
- Mycket låg .... ☐

Ej förskrivit detta... ☐

12 Under de tre senaste månaderna, hur anser du att din förskrivning av **lugnande/sömnmedel** varit i förhållande till dina patienters medicinska behov?

- Mycket hög .... ☐
- Ganska hög .... ☐
- Varken/eller .... ☐
- Ganska låg .... ☐
- Mycket låg .... ☐

Ej förskrivit detta... ☐

13 Under de tre senaste månaderna, hur anser du att din förskrivning av **antipsykotika** varit i förhållande till dina patienters medicinska behov?

- Mycket hög .... ☐
- Ganska hög .... ☐
- Varken/eller .... ☐
- Ganska låg .... ☐
- Mycket låg .... ☐

Ej förskrivit detta... ☐

Din bakgrund

14 Vad har du för utbildning? Flera val kan göras.

- Specialist allmänmedicin.. ☐
- Specialist något annat...... ☐
- ST allmänmedicin............ ☐
- ST något annat .... ☐
- Legitimerad, ej ST .... ☐
- AT-läkare.......................... ☐
- Ej legitimerad, ej AT......... ☐

15 Vilket år erhöll du läkarlegitimation?

Svar ....

16 Är du...

- Kvinna .... ☐
- Man .... ☐

17 Är denna vårdcentral din ordinarie arbetsplats?

- Ja .... ☐
- Nej .... ☐

18 Kan du remittera patienter till samtalsterapi på vårdcentralen?

- Ja .... ☐
- Nej .... ☐

19 Kan du remittera patienter till psykiatriker som finns på vårdcentralen?

- Ja .... ☐
- Nej .... ☐

20 Har du under det senaste kvartalet varit med på information från läkemedelsindustrin?

- Ja .... ☐
- Nej .... ☐

21 Övriga kommentarer? ....

....

Tack för din medverkan!
