# Supplementary material for: Attitudes and behaviour towards psychotropic drug prescribing in Swedish primary care: a questionnaire study
Source: BMC Fam Pract. 2019 Jan 5;20:4. doi: 10.1186/s12875-018-0885-4 (PMC6321667; doi:10.1186/s12875-018-0885-4)
Supplement: Supplementary file 2 — Questionnaire translation. With names of variables, original text and English translation. (PDF 52 kb) [file 12875_2018_885_MOESM2_ESM.pdf]

Questionnaire with original text and translation

Supplementary file to “Attitudes and behaviour towards psychotropic drug prescribing in Swedish primary care: a questionnaire study” by Svensson, Hedenrud & Wallerstedt 2018.

. . . .

Vad tycker du?

I intervjuer med vårdcentralsläkare om psykofarmaka (antidepressiva läkemedel, lugnande/sömnmedel och antipsykotika) fick vi en rad förslag på faktorer som kan påverka förskrivningen. Vi skulle vilja veta din åsikt om dessa, och hur du ser på din egen förskrivning!

What do you think?

In interviews with primary care physicians about psychotropics (antidepressant drugs, anxiolytics/hypnotics and antipsychotics), we got a number of proposals of factors that may influence prescribing. We would like to know your opinion on these, and how you regard your own prescribing!

1. `a01latta`
   Vad tycker du är lättast: att sätta **in** eller sätta **ut** psykofarmaka? Svar: Sätta in mycket lättare (1), Sätta in något lättare (2), Ingen skillnad (3), Sätta ut något lättare (4), Sätta ut mycket lättare (5)

   Which do you find easier: **starting** or **stopping** prescribing psychotropics? Replies: Starting much easier (1), Starting somewhat easier (2), No difference (3), Stopping somewhat easier (4), Stopping much easier (5)

2. `a02socek`
   Upplever du att de problem du behandlar med psykofarmaka är sociala/ekonomiska, snarare än medicinska? Svar: Ja, mycket ofta (1), Ja, ganska ofta (2), Varken/eller (3), Nej, ganska sällan (4), Nej, mycket sällan (5)

   In your experience, are the problems you treat with psychotropics more social/economic, rather than medical? Replies: Yes, very often (1), Yes, quite often (2), Neither one nor the other (3), No, quite rarely (4), No, very rarely (5)

3. `a03kolle`
   Hur tycker du det är att ändra en kollegas ordination av psykofarmaka? Svar: Mycket svårt (1), Ganska svårt (2), Varken/eller (3), Ganska lätt (4), Mycket lätt (5)

   How do you feel about changing a colleague’s psychotropic prescription? Replies: Very difficult (1), Somewhat difficult (2), Neither one nor the other (3), Quite easy (4), Very easy (5)

4. `a04pdyra`
   Tror du att dina patienter uppfattar dyra psykofarmaka som mer eller mindre effektiva än billiga? Svar: Mycket mer effektiva (1), Något mer effektiva (2), Ingen skillnad (3),

Något mindre effektiva (4), Mycket mindre effektiva (5)

Do you believe your patients perceive expensive psychotropics as being more or less effective than cheap ones? Replies: Much more effective (1), Somewhat more effective (2), Neither one nor the other (3), Somewhat less effective (4), Much less effective (5)

5. `a05bemot`
   Hur upplever du att sjukvårdspersonal bemöter patienter som står på psykofarmaka, jämfört med andra patienter? Svar: Mycket bättre (1), Något bättre (2), Ingen skillnad (3), Något sämre (4), Mycket sämre (5)

   In your experience, how do health care staff behave towards patients who use psychotropics, compared to their behaviour towards other patients? Replies: Much better (1), Somewhat better (2), Neither one nor the other (3), Somewhat worse (4), Much worse (5)

6. `a06nyskr`
   När du arbetar i primärvården, skriver du då ut psykofarmaka som är nya på marknaden? Svar: Ja, mycket ofta (1), Ja, ganska ofta (2), Varken/eller (3), Nej, ganska sällan (4), Nej, mycket sällan (5)

   When working in primary care, do you prescribe psychotropics that have recently appeared on the market? Replies: Yes, very often (1), Yes, quite often (2), Neither one nor the other (3), No, quite rarely (4), No, very rarely (5)

7. `a07nyeff`
   Anser du att nya psykofarmaka överlag är mer eller mindre effektiva än gamla? Svar: Mycket mer effektiva (1), Något mer effektiva (2), Ingen skillnad (3), Något mindre effektiva (4), Mycket mindre effektiva (5)

   In your opinion, are new psychotropics more effective than older ones? Replies: Much more effective (1), Somewhat more effective (2), Neither one nor the other (3), Somewhat less effective (4), Much less effective (5)

8. `a08apods`
   Om din patient har Apodos, händer det då att du förnyar flera recept samtidigt utan att värdera varje enskild ordination? Svar: Ja, mycket ofta (1), Ja, ganska ofta (2), Varken/eller (3), Nej, ganska sällan (4), Nej, mycket sällan (5)

   If your patient has dose-dispensed drugs, do you ever repeat several prescriptions at the same time, without assessing each individual prescription? Replies: Yes, very often (1), Yes, quite often (2), Neither one nor the other (3), No, quite rarely (4), No, very rarely (5)

9. `a09sampf`
   Vid lindrig psykiatrisk sjukdom, vilken behandlingsform anser du mest lämplig: samtalsterapi (S) eller psykofarmaka (P)? Svar: (1) S mycket lämpligare (1), S något lämpligare (2), Ingen skillnad (3), P något lämpligare (4), P mycket lämpligare (5)

   In mild psychiatric disease, what kind of treatment do you consider most suitable: psychotherapy (PT) or psychotropic drugs (PD)? Replies: PT much more suitable (1), PT somewhat more suitable (2), No difference (3), PD somewhat more suitable (4), PD much more suitable (5)

10. `a10varia`
    Vid samma symtombild, hur mycket tror du förskrivningen av psykofarmaka varierar mellan olika vårdcentraler? Svar: Den varierar mycket (1), Den varierar något (2), Den varierar försumbart (3)

Keeping symptoms constant, how much do you suppose psychotropic prescribing varies between different GP practices? Replies: It varies considerably (1), It varies somewhat (2), It varies negligibly (3)

11. `f11antdp`
    Under de tre senaste månaderna, hur anser du att din förskrivning av **antidepressiva läkemedel** varit i förhållande till dina patienters medicinska behov? Svar: Mycket hög (1), Ganska hög (2), Varken/eller (3), Ganska låg (4), Mycket låg (5), Ej förskrivit detta (0)

    Over the last three months, how would you describe the level of your prescribing of **antidepressants**, in relation to the medical needs of your patients? Replies: Very high (1), Somewhat high (2), Neither one nor the other (3), Somewhat low (4), Very low (5), Have not prescribed (0)

12. `f12anxly`
    Under de tre senaste månaderna, hur anser du att din förskrivning av **lugnande/sömnmedel** varit i förhållande till dina patienters medicinska behov? Svar: Mycket hög (1), Ganska hög (2), Varken/eller (3), Ganska låg (4), Mycket låg (5), Ej förskrivit detta (0)

    Over the last three months, how would you describe the level of your prescribing of **anxiolytics/hypnotics**, in relation to the medical needs of your patients? Replies: Very high (1), Somewhat high (2), Neither one nor the other (3), Somewhat low (4), Very low (5), Have not prescribed (0)

13. `f13anpsy`
    Under de tre senaste månaderna, hur anser du att din förskrivning av **antipsykotika** varit i förhållande till dina patienters medicinska behov? Svar: Mycket hög (1), Ganska hög (2), Varken/eller (3), Ganska låg (4), Mycket låg (5), Ej förskrivit detta (0)

    Over the last three months, how would you describe the level of your prescribing of **antipsychotics**, in relation to the medical needs of your patients? Replies: Very high (1), Somewhat high (2), Neither one nor the other (3), Somewhat low (4), Very low (5), Have not prescribed (0)

14. `utb`
    Vad har du för utbildning? Flera val kan göras. Svar: Specialist allmänmedicin; Specialist något annat; ST allmänmedicin; ST något annat; Legitimerad, ej ST; AT-läkare; Ej legitimerad, ej AT

    What is your education? Several choices are possible. Replies: Specialist in General Practice; Specialist in other; Specialisation programme in General Practice; Specialisation programme in other; Registered, no specialisation programme; Pre-registration physician (intern); Pre-registration (not intern)

15. `aarleg`
    What year did you obtain full medical registration?

    Vilket år erhöll du läkarlegitimation?

16. `kon`
    Är du… Svar: Kvinna; Man

    Are you a… Replies: Woman; Man

17. `ordarb`
    Är denna vårdcentral din ordinarie arbetsplats? Svar: Ja; Nej

    Is this GP practice your regular workplace? Replies: Yes; No

18. `remsamt`
    Kan du remittera patienter till samtalsterapi på vårdcentralen? Svar: Ja; Nej

    Can you refer patients to psychotherapy at your GP practice? Replies: Yes; No

19. `rempsyk`
    Kan du remittera patienter till psykiatriker som finns på vårdcentralen? Svar: Ja; Nej

    Can you refer patients to a psychiatrist present at your GP practice? Replies: Yes; No

20. `induinfo`
    Har du under det senaste kvartalet varit med på information från läkemedelsindustrin? Svar: Ja; Nej

    Have you, in the last quarter of a year, attended information from the pharmaceutical industry? Replies: Yes; No

21. `...`
    Övriga kommentarer? Svar: fritext

    Other comments? Replies: free text
